# BioSunMS: a plug-in-based software for the management of patients information and the analysis of peptide profiles from mass spectrometry

**DOI:** 10.1186/1472-6947-9-13

**Published:** 2009-02-17

**Authors:** Yuan Cao, Na Wang, Xiaomin Ying, Ailing Li, Hengsha Wang, Xuemin Zhang, Wuju Li

**Affiliations:** 1 Center of Computational Biology, Beijing Institute of Basic Medical Sciences, Taiping Road 27, Haidian district, Beijing 100850, PR China; 2 National Center of Biomedical Analysis, Taiping Road 27, Haidian district, Beijing 100850, PR China

## Abstract

**Background:**

With wide applications of matrix-assisted laser desorption/ionization time-of-flight mass spectrometry (MALDI-TOF MS) and surface-enhanced laser desorption/ionization time-of-flight mass spectrometry (SELDI-TOF MS), statistical comparison of serum peptide profiles and management of patients information play an important role in clinical studies, such as early diagnosis, personalized medicine and biomarker discovery. However, current available software tools mainly focused on data analysis rather than providing a flexible platform for both the management of patients information and mass spectrometry (MS) data analysis.

**Results:**

Here we presented a plug-in-based software, BioSunMS, for both the management of patients information and serum peptide profiles-based statistical analysis. By integrating all functions into a user-friendly desktop application, BioSunMS provided a comprehensive solution for clinical researchers without any knowledge in programming, as well as a plug-in architecture platform with the possibility for developers to add or modify functions without need to recompile the entire application.

**Conclusion:**

BioSunMS provides a plug-in-based solution for managing, analyzing, and sharing high volumes of MALDI-TOF or SELDI-TOF MS data. The software is freely distributed under GNU General Public License (GPL) and can be downloaded from http://sourceforge.net/projects/biosunms/.

## Background

With wide applications of MALDI-TOF MS and SELDI-TOF MS in biomedical studies, more and more large-scale MS datasets have being obtained [1-6]. How to extract useful information from these datasets not only needs a variety of statistical analysis, but also asks for patients information. Thus, an efficient and flexible software is needed to comprehensively handle so much information and so many analytical tools. Up to now, some software has been developed for MS data analysis. For example, the database built by Titulaer [7] can analyze high-throughput MS data from MALDI-TOF MS measurements. But it can not manage and analyze the clinical information related to the MS experimental results. In addition, Josep et al have developed a laboratory information management system for characterizing and standardizing the entire sample collection and serum preparation process [8]. The data processing and analysis were done in MATLAB [9] and GeneSpring [10]. Recently, Marc Sturm group presented a software framework, OpenMS, for rapid application development in mass spectrometry [11]. However, it is not specific for the analysis of MALDI-TOF or SELDI-TOF MS. Furthermore, there are many other projects for MS data processing and analysis e.g. MapQuant [12], MASPECTRAS [13], SpecArray [14], msInspect [15] and MZMine [16]. But few projects try to emphasize both the management of patients information and MALDI-TOF or SELDI-TOF MS-related statistical analysis. Moreover, none of them provides an integrated solution for clinical researchers without any knowledge in programming, as well as a plug-in architecture platform with the possibility for developers to add or modify functions without need to recompile the entire application.

Here we developed a flexible and compact software, BioSunMS, for MALDI-TOF or SELDI-TOF MS-based clinical proteomics study. The name BioSunMS was coined by the combination of BioSun and MS (mass spectroemtry), in which BioSun stands for the comprehensive bioinformatics software developed by our center [17]. BioSunMS was designed to support decision-making and allow patients information and spectra data to be stored, managed, processed, and analyzed using the Rich Client Platform (RCP) [18] from Eclipse [19]. The BioSunMS software had been tested with MS files of serum samples from patients with lung cancer and a control group.

## Implementation

### System architecture

BioSunMS provides a relational database and client-server architecture suitable for multi-user workgroups. It is a RCP application extending the Eclipse framework. It was developed in Java, and provided a domain-specific platform where the plug-ins can be integrated. End-users can select related features from the BioSunMS plug-ins freely.

**Figure 1:**
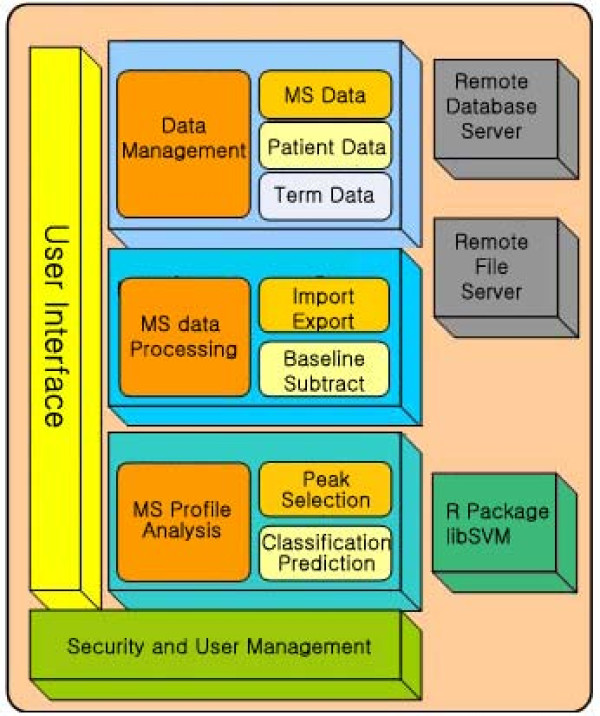
**The main components of the BioSunMS**. Four components are provided in BioSunMS system, which are Security and User Management, Data Management, MS data Processing, and MS profile Analysis, respectively.

BioSunMS system includes four functional modules, namely, data management, spectrum processing, MS profile analysis and security module (Figure 1). The data management module provides a robust, client-server, relational database system for the management of patients information and MS data. Data are primarily stored in a relational database MySQL, while some raw data is stored on the file system (mainly to provide backup for MS data). The spectrum processing module performs spectrum import, spectrum export, and related analysis such as calibration, normalization and peak detection [20]. The MS profile module is designed for sample classification and identification of potential biomarkers. Background computations are mostly done by R package and libSVM [21]. BioSunMS has a security system for protecting the data. Access to any data can be controlled. Access is granted via user groups rather than individual users. The main features of BioSunMS can be found in Table 1.

**Table 1:** The main features of BioSunMS software.

Feature Item	Feature description
Database	MySQL based relational database system for patients information and MS data.
Preprocessing	Baseline subtraction and smoothing; Normalization; Automatic or manual peak detection; Peak alignment
MS format	Support common MS format, such as mzML, mzXML, mzData,
Visualization	Graphics display for MS raw data and MS peaks
Statistics or procedures	t-test for feature selection; SVM or kNN-based model construction;
ROC	The ROC curve for the evaluation of model performance
Security	Client-server architecture for multi-user workgroups
Scalability	Plug-in framework for future development

### User Interface

BioSunMS is built using Standard Widget Toolkit (SWT). Created as part of the Eclipse project, SWT allows developers to build efficient and portable applications. In contrast to Swing/AWT (which provide their own graphical environment), SWT has the same look and feel of the operating system on which the application runs. AWT/Swing components can be wrapped in SWT, and this feature is utilized in BioSunMS to integrate Java components built on these toolkits [22]. In an Eclipse RCP application, the user interface is composed of five main graphical units, namely, View, Editor, Perspective, Menu and Wizard. A View is a graphical window to provide information for the users. An Editor is another type of window to let the users edit and save data. A perspective is a visual container for a set of views and content editors. An example in BioSunMS is the Spectral Perspective, which displays Views, Editors, and Menu options for spectral data. Wizards are used to guide users to finish particular object through a sequential set of tasks. The internal placement and size of components within a perspective are not fixed but can be changed at the user preference and is saved between sessions.

### Workflow

**Figure 2:**
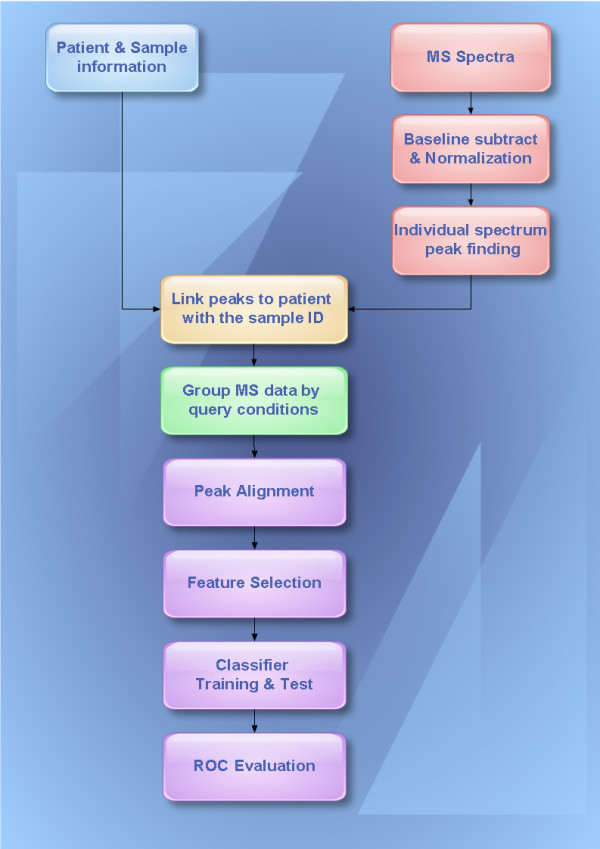
**The workflow charts of the BioSunMS**. To construct model for patients classification using peptide profiles generated by MALDI-TOF MS or SELDI-TOF MS, following steps are suggested. The first step is to input patients information, then preprocessing of MS spectra data for each patient, linking MS peaks to each patient through sample ID, peak alignment and creation of data matrix, t-test based feature selection and SVM-based model construction. Finally, the ROC curve was used to evaluate the performance of the model.

To access the BioSunMS server at client side, users must first log on with an authorized username and a given password. Users could also log on for analyzing spectral data without connecting to the server, when their computers are offline. The workflow of BioSunMS was illustrated in Figure 2, which was divided into four main parts as follows.

### Enter new records

The first step in the workflow is to input patients general information and laboratory test results. To add a new record, click on the top-right button "Append" in the toolbar. A form will be displayed to enter data according to the module. Fields include equipment, sample ID, gender, age, specimen group and so on. Marked fields must be specified. Users could group samples with the Query View.

### Import spectra to the BioSunMS

**Figure 3:**
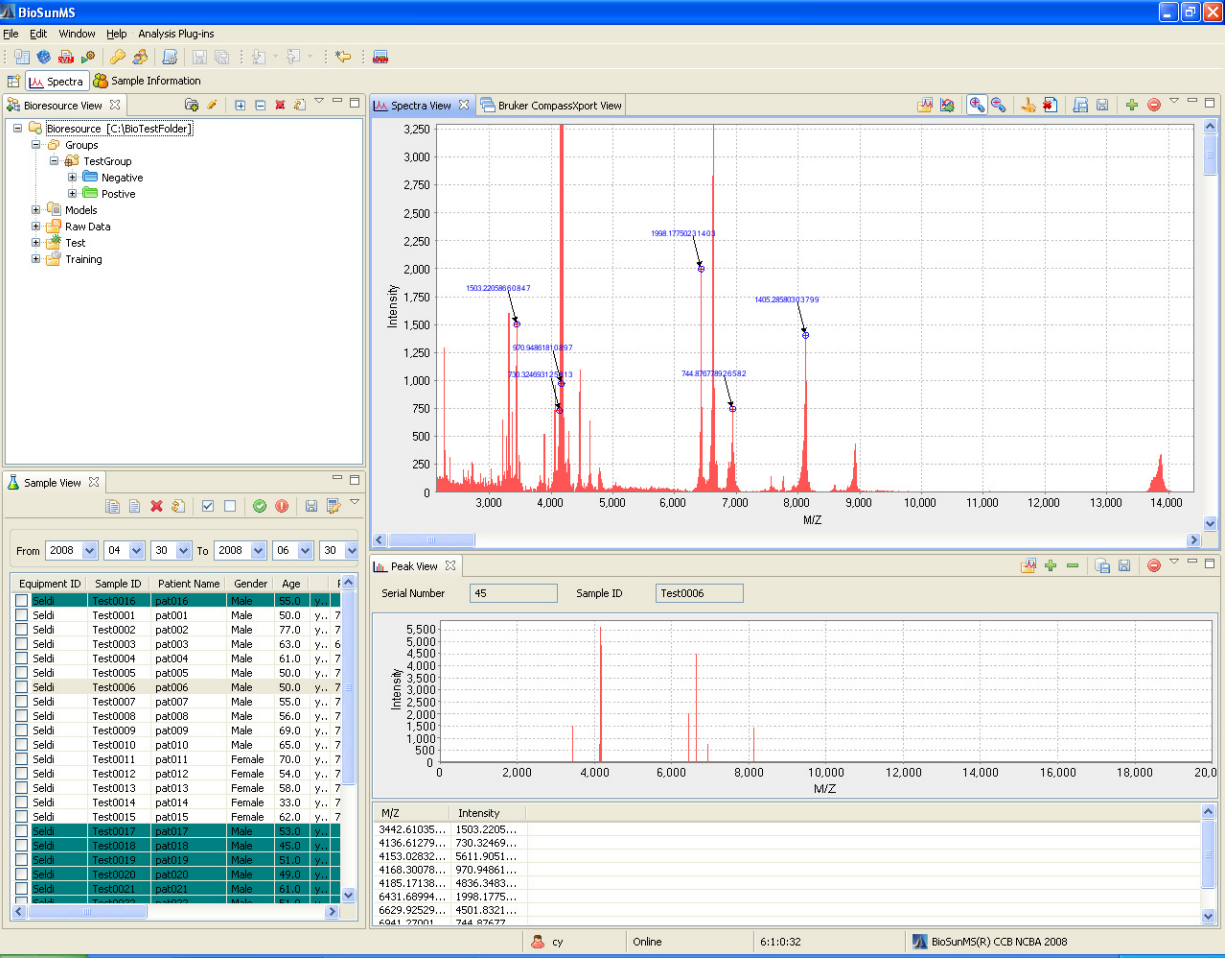
**The interface for MS data display**. The preprocessing of MS data for a particular patient was demonstrated, which included baseline subtraction, normalization, and automatic or manual peak extraction.

The second step in the workflow is to import spectral files to the BioSunMS. After importing a spectral file, BioSunMS uses the Bioconductor PROcess package developed by Li [23] to process the spectral data. The PROcess package contains a collection of functions for removing baseline drifts, detecting peaks and aligning peaks for a set of biomarkers. In the Spectra View which uses the JFreeChart package [24] for visualization of spectral information, users can identify or erase the peaks in a spectrum manually. Labeled peaks are shown in the Peak View. After data processing, the peak list can be saved to the database corresponding to the sample ID (Figure 3). Users can upload the "raw" spectral files to the ftp server for backup.

### Group spectra into folders

**Figure 4:**
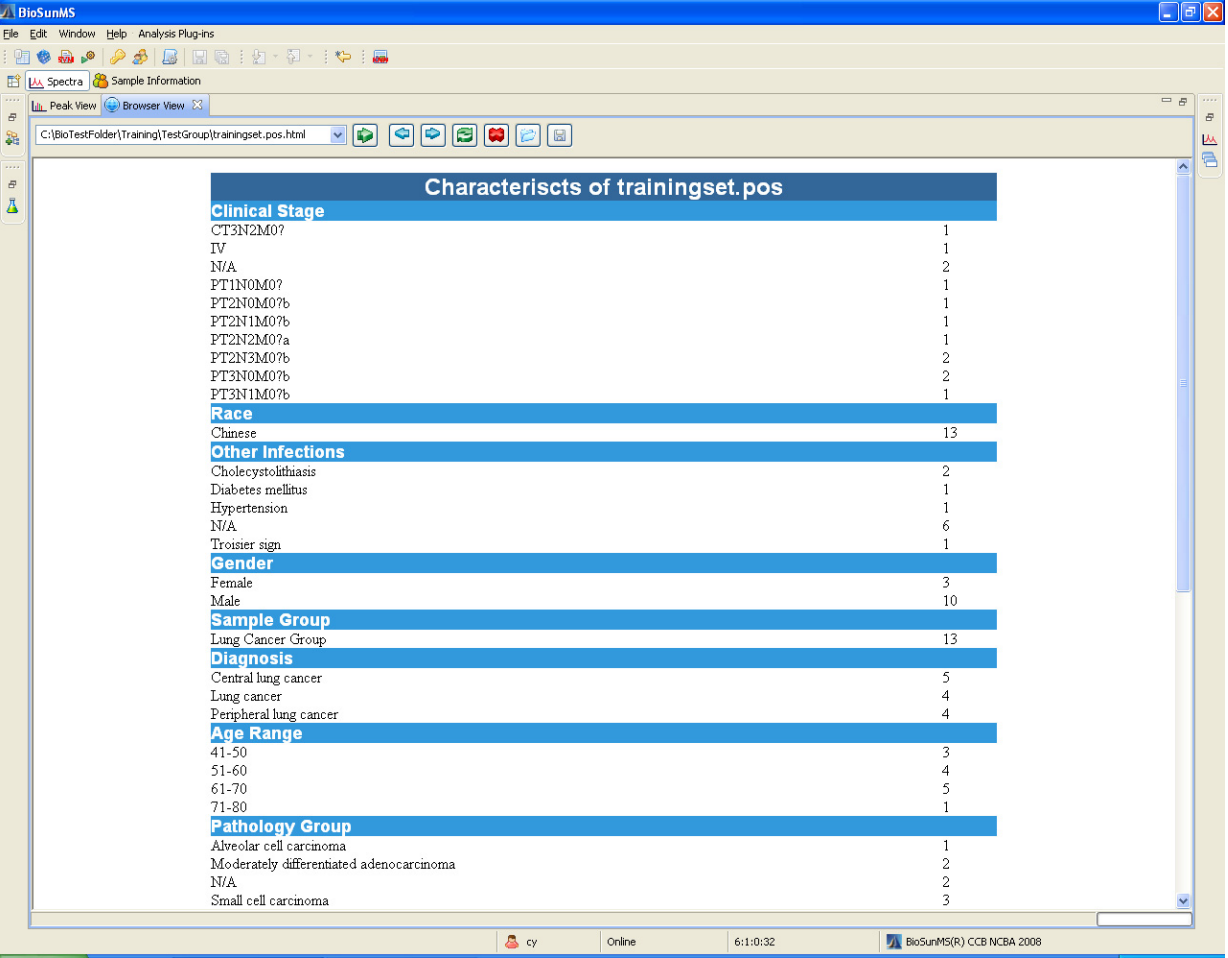
**Summary of patients information**. The patients characteristics were provided in a web page, which included clinical stage, age range, pathology diagnosis, and etc.

The third step in the workflow is to group spectral files into the Bioresource folder. To analyze data, spectra must be grouped together into a folder. There are many ways to organize spectra by querying the database for spectra meeting desired criteria, such as research group, user, sample state, sample type, patient and characteristic description. Users can also define the condition by selecting a series of fields to be used to separate spectra into groups for classification and prediction. After grouping, a web page with a table of characteristics of patients can be created (Figure 4).

### Apply analysis algorithms

**Figure 5:**
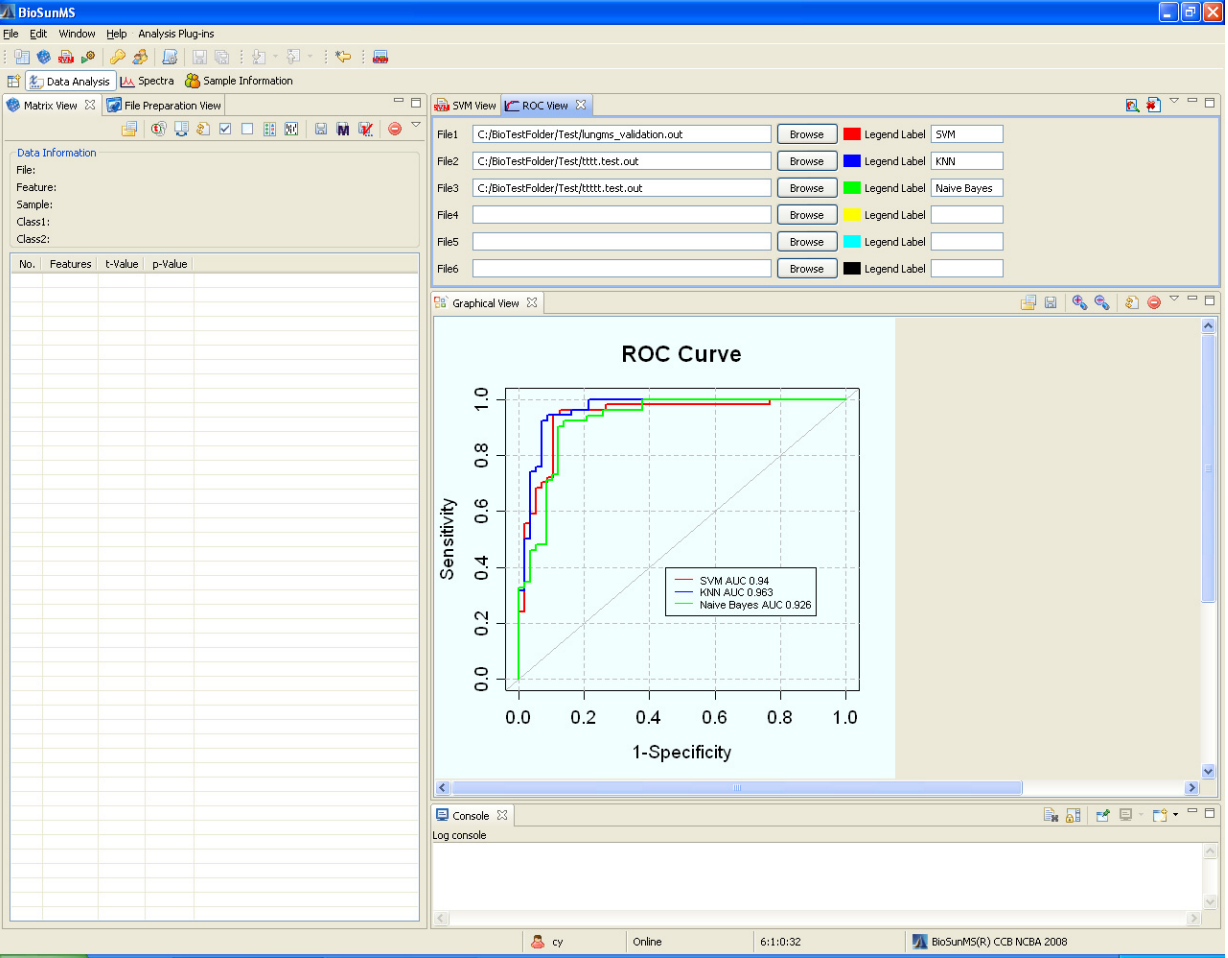
**The screen shot of ROC curve**. The ROC curve is used to evaluate the performance of model. The demonstrated example provides the ROC curves for classifiers constructed from SVM, KNN and Naive Bayes methods, respectively, and the correspondent areas under the ROC Curve are 0.94, 0.963 and 0.926.

The fourth step in the workflow is to analyze the experimental results for identification of potential biomarkers and early diagnosis. BioSunMS provides the Wizard for general users, including extraction of MS peaks, construction of m/z matrix, sample classification and sample prediction. Advanced users can process and analyze the MS data in the Data Analysis Perspective. After building a Support Vector Machine (SVM) model on the training dataset and make prediction on the test dataset, BioSunMS uses a Receiver Operation Characteristic (ROC) curve to assess the models performance (Figure 5).

## Results and discussion

BioSunMS is a RCP application through bringing the power of lab's network to access and manage a large amount of information. It can be installed on Windows or Linux system. It allows researchers to enter patient data in a customizable template and group the data by queries. It has a security system for protecting data. Access is granted via user groups rather than individual users.

### Security and access

BioSunMS is a network-based system like an Intranet. It can be accessed from all computers from the same local network. It can even be accessed through the internet. Therefore, data access and management can be password protected. The system always stores actions made by any users. The administrator can check the action history at any time.

From the User and Group Management View, BioSunMS administrator can add or edit the user and the group. If a user group has been permitted to access a kind of data, any users belonging to the group are permitted to read the data in the Sample View. Any user who doesn't belong to the user group will not be permitted to read the data in the Sample View. Any user can create a new record, and modify a record which belongs to the same user group. However, only administrators can create new users and user groups. By default a new record is accessible to all users. To restrict access to a record, assign that record to the desired user group.

### Sample and clinical information

Recording detailed information on collection, processing and storage of samples is crucial both for efficient reporting on biomedical study and for subsequent data analysis[25]. Many patients and sample related variables, such as gender, age, genetic factors, drug treatment, and symptom, dietary, and family history are particularly important in the context of clinical proteomics study. BioSunMS provides an efficient link between sample data and experiment results. Users can define the condition by selecting a series of fields to be used to automatically segregate spectra into groups. For example, in a typical profiling study, a condition might consist of the array type, sample type, different subjects (e.g. healthy and diseased), clinical laboratory result and diagnosis. After grouping the data by condition, BioSunMS will create a table with characteristics of the group of patients information (Figure 4). Finally, the data produced by the above steps can be exported for further processing outside of BioSunMS system using the export function provided by Bioresource View.

### Pre-processing and visualization

BioSunMS is useful for the users with the need to store, process and analyze MALDI-TOF or SELDI-TOF MS data. It supports general mass spectrometry file format, such as mzML, mzXML, mzData and CSV. For mzML data format standard released by the HUPO-PSI and Institute for Systems Biology in June 2008, BioSunMS reads and writes it using a package of ProteomeCommons.org IO Framework [26] and ProteoWizard[27]. Currently, the majority of instruments store mass spectra in vendor specific formats. Users can convert raw file to universal file, for example, mzXML format using the converter programs provided by the vendors. At present, we have developed a plug-in which integrates CompassXport[28] for converting raw file of Bruker Corporation[29] to mzXML. Other plug-ins for format converting is under development.

BioSunMS provides basic visualization tools and advanced processing algorithms. The method and related procedure, BioConductor PROcess was used for baseline subtraction, smoothing, normalization and peaks identification. Our data visualization module aims to support analysts in finding interesting peaks in spectra, selecting them for further analysis and visualizing the selected peaks by automatic or manual detection.

### Sample classification and sample prediction

The analysis of several spectra coming from biological samples belonging to different subjects (e.g. healthy and diseased) focuses on identifying discriminant values in spectra related to diseases. The BioSunMS allows users to select features using t-test, construct models using SVM, and predict new samples by the wizard with the default parameters. Users can compare the generalization performance of a range of classifiers by plotting their performance on the test set in ROC curve. BioSunMS also provides heat maps and hierarchical clustering. In the future, we will gradually incorporate classification system Tclass [30] and sample class discovery system SamCluster [31] into BioSunMS, which were developed by our center for gene expression profile-based analysis.

### R package

BioSunMS uses the R statistical programming language and some third part packages for processing and analysis of MS data. We implemented the communication between the BioSunMS and the R package with the class ncbaSpanker.System.R.RPackage. This class provides some methods for executing R scripts and commands. All communication with R language takes place via the file system. In general, BioSunMS writes a R script, makes the R system execute the script file, and output the results. Then BioSunMS reads an R output file to retrieve the results. The RPackage class hides all the details of communication with R. So users with little knowledge about R language can accomplish their task easily. Because many programs for gene expression profile-based analysis have been provided in R package, and some algorithms can be directly used in peptide profiling analysis, we use R package as the background computation. In addition, many packages for MS data analysis and visualization are also provided, for example, GALGO[32], caMassClass [33] and so on. We will incorporate them into BioSunMS system in the future.

### Development environment

Here we want to emphasize an important characteristic of BioSunMS. It can not only be used as the standard-alone program, but also can be a framework for developers. By taking full advantage of the editing and visualization components provided by Eclipse, the developers can focus entirely on the problems at hand. Moreover, BioSunMS was implemented in Eclipse platform, which made it more flexible and easier to adopt an algorithm as a plug-in. For example, we have successfully developed a plug-in, cn.org.biosun.knn, for sample classification using k-nearest neighbour (kNN) method.

## Conclusion

BioSunMS is a plug-in based and flexible software for MS data management and analysis. It integrates patients information and MS data storage, process, sample classification and sample prediction into a single, user-friendly workbench. The project provides an alternative solution to analyze high throughput MS data from MALDI-TOF or SELDI-TOF.

The future for BioSunMS holds much potential with many plug-ins in development, including Web service, more statistical methods and machine learning algorithms, such as GA, Decision-Tree, Naïve Bayes method, and sample class discovery. There is also ongoing work for improving the plug-ins which allows researchers to enter patients data (i.e., past history, patient history, physical examination, social history, and family history data) in a structured way using a customizable domain model. Another major feature in development is online updates for plug-in from the BioSunMS update server. The current status of the BioSunMS development can be viewed at the BioSunMS website.

## Availability and requirements

**Project name**: BioSunMS

**Project home page**: http://ccb.bmi.ac.cn/biosunms/ or http://sourceforge.net/projects/biosunms/

**Operating system(s)**: Windows, Linux

**Programming language**: Java, R, SQL

**Other requirements**: SUN JVM 1.5 or higher, R Package 2.5.1 or higher, MySql 5.0 or higher

**License**: GNU GPL

**Any restrictions to use by non-academics**: Contact authors

## Abbreviations

RCP: Rich Client Platform; SWT: Standard Widget Toolkit; MS: Mass Spectrometry; MALDI-TOF MS: Matrix-Assisted Laser Desorption/Ionization Time-Of-Flight Mass Spectrometry; SELDI-TOF MS: Surface-Enhanced Laser Desorption/Ionization Time-Of-Flight Mass Spectrometry; ROC: Receiver Operation Characteristic; SVM: Supported Vector Machines; kNN: k-Nearest Neighbour

## Competing interests

The authors declare that they have no competing interests.

## Authors' contributions

YC designed and developed the system. XY, NW, AL and HW did the conceptual design of the system and gave some detailed technique supports. XZ and WL conceived the project, performed overall supervision and coordination. All authors read and approved the final manuscript.

## Pre-publication history

The pre-publication history for this paper can be accessed here:

http://www.biomedcentral.com/1472-6947/9/13/prepub
